# Supplementary material for: Prediction of Persistent Tumor Status in Nasopharyngeal Carcinoma Post-Radiotherapy-Related Treatment: A Machine Learning Approach
Source: Cancers (Basel). 2024 Dec 31;17(1):96. doi: 10.3390/cancers17010096 (PMC11720740; doi:10.3390/cancers17010096)
Supplement: Supplementary file 1 [file cancers-17-00096-s001.zip › cancers-3371114-supplementary.pdf]

Supplementary Materials: The following supporting information can be downloaded at: https://www.mdpi.com/article/10.3390/cancers17010096/s1,

Table S1: Diagnostic performance of radiomic features.

		RF_{50}				RF_{60}				RF_{Diff}			
			95% CI				95% CI				95% CI		
Matrix	Feature	AUC ± SD	LB	UB	Sig	AUC ± SD	LB	UB	Sig	AUC ± SD	LB	UB	Sig
Conventional	SUV_{Max}	.614 ± .064	.489	.739	.073	.614 ± .064	.489	.739	.073	-	-	-	-
	MTV	.547 ± .066	.417	.678	.476	.486 ± .068	.353	.618	.833	.577 ± .067	.445	.709	.251
	TLG_{Max}	.595 ± .063	.472	.718	.129	.553 ± .062	.432	.674	.390	.632 ± .063	.509	.755	**.036**
	SUV_{Mean}	.601 ± .064	.476	.727	.114	.603 ± .064	.477	.728	.109	.596 ± .064	.471	.721	.131
	TLG_{Mean}	.595 ± .062	.473	.718	.126	.549 ± .062	.428	.670	.428	.642 ± .062	.520	.765	**.023**
First Order	10th Percentile	.613 ± .064	.488	.738	.077	.612 ± .064	.487	.736	.078	.621 ± .062	.500	.742	.051
	90th Percentile	.597 ± .064	.471	.722	.131	.605 ± .065	.479	.732	.104	.559 ± .066	.431	.688	.368
	Energy	.615 ± .059	.499	.730	.052	.574 ± .060	.456	.691	.219	.681 ± .057	.569	.793	**.002**
	Entropy	.409 ± .062	.288	.530	.142	.402 ± .060	.284	.520	.104	.620 ± .061	.501	.738	**.048**
	Interquartile Range	.558 ± .064	.433	.683	.365	.587 ± .065	.460	.714	.178	.628 ± .061	.508	.748	**.036**
	Mean Absolute Deviation	.563 ± .064	.439	.688	.320	.587 ± .066	.458	.716	.185	.607 ± .062	.486	.729	.084
	Median	.604 ± .064	.478	.729	.105	.603 ± .064	.478	.728	.106	.569 ± .064	.444	.694	.278
	Minimum	.614 ± .064	.489	.739	.074	.613 ± .064	.488	.738	.077	.608 ± .064	.483	.732	.090
	Range	.614 ± .064	.490	.739	.072	.617 ± .063	.492	.741	.066	.608 ± .064	.483	.732	.090
	Robust Mean Absolute Deviation	.551 ± .064	.426	.675	.424	.589 ± .065	.461	.716	.174	.580 ± .064	.454	.706	.214
	Root Mean Squared	.600 ± .064	.474	.727	.118	.602 ± .064	.476	.728	.111	.597 ± .064	.471	.723	.132
	Total Energy	.615 ± .059	.499	.730	.052	.574 ± .060	.456	.691	.219	.681 ± .057	.569	.793	**.002**
	Uniformity	.569 ± .063	.446	.692	.274	.586 ± .061	.467	.705	.158	.544 ± .068	.411	.677	.518
	Standard Deviation	.570 ± .064	.445	.695	.271	.592 ± .065	.463	.720	.161	.611 ± .061	.491	.731	.071
	Variance	.570 ± .064	.445	.695	.271	.592 ± .065	.463	.720	.161	.605 ± .061	.485	.725	.087

	Skewness	.560 ± .062	.439	.681	.331	.603 ± .059	.488	.719	.080	.474 ± .066	.344	.604	.695
	Kurtosis	.590 ± .061	.471	.709	.137	.593 ± .060	.476	.709	.120	.604 ± .062	.482	.726	.094
Shape	Elongation	.392 ± .065	.265	.520	.098	.420 ± .061	.302	.539	.188	.539 ± .067	.407	.672	.560
	Flatness	.451 ± .062	.329	.574	.438	.486 ± .060	.369	.602	.808	.642 ± .064	.517	.767	.026
	Least Axis Length	.570 ± .065	.442	.698	.283	.536 ± .064	.410	.662	.579	.552 ± .071	.412	.691	.467
	Major Axis Length	.598 ± .065	.472	.725	.128	.531 ± .064	.406	.657	.622	.611 ± .063	.488	.735	.077
	Maximum 2D Diameter Column	.586 ± .066	.456	.716	.193	.523 ± .068	.389	.657	.734	.606 ± .063	.483	.728	.090
	Maximum 2D Diameter Row	.518 ± .065	.392	.645	.776	.491 ± .063	.368	.614	.883	.620 ± .059	.505	.734	.041
	Maximum 2D Diameter Slice	.564 ± .063	.440	.688	.312	.517 ± .062	.396	.638	.780	.622 ± .065	.495	.750	.061
	Maximum 3D Diameter	.598 ± .065	.471	.725	.130	.526 ± .064	.401	.652	.680	.661 ± .057	.548	.773	.005
	Mesh Volume	.547 ± .066	.417	.677	.478	.486 ± .067	.354	.618	.835	.577 ± .067	.445	.708	.253
	Minor Axis Length	.505 ± .068	.372	.637	.943	.486 ± .067	.353	.618	.830	.504 ± .068	.371	.636	.954
	Sphericity	.371 ± .062	.250	.493	.039	.416 ± .066	.287	.544	.198	.606 ± .063	.483	.730	.092
	Surface Area	.565 ± .067	.434	.696	.330	.507 ± .067	.376	.638	.920	.606 ± .067	.474	.737	.117
	Surface Volume Ratio	.529 ± .065	.402	.655	.657	.546 ± .064	.420	.672	.472	.529 ± .068	.396	.661	.671
	Voxel Volume	.547 ± .066	.417	.678	.476	.486 ± .068	.353	.618	.833	.577 ± .067	.445	.709	.251
MUCM	Autocorrelation	.394 ± .062	.272	.515	.086	.407 ± .062	.287	.528	.133	.517 ± .063	.393	.641	.785
	Cluster Prominence	.393 ± .067	.262	.524	.109	.436 ± .065	.309	.563	.323	.449 ± .068	.316	.581	.448
	Cluster Shade	.468 ± .063	.346	.591	.609	.574 ± .061	.455	.694	.224	.452 ± .061	.332	.572	.433
	Cluster Tendency	.384 ± .064	.259	.509	.069	.427 ± .064	.301	.553	.256	.523 ± .064	.398	.649	.717
	Contrast	.405 ± .065	.279	.532	.141	.461 ± .067	.329	.593	.559	.519 ± .063	.396	.642	.761
	Correlation	.396 ± .058	.283	.510	.074	.437 ± .060	.320	.555	.295	.505 ± .061	.385	.626	.931

	Difference Average	.398 ± .064	.273	.524	.112	.460 ± .067	.329	.592	.556	.577 ± .063	.453	.701	.224
	Difference Entropy	.422 ± .062	.301	.543	.207	.420 ± .058	.306	.533	.166	.543 ± .063	.419	.666	.501
	Difference Variance	.430 ± .066	.301	.559	.290	.460 ± .067	.329	.591	.547	.451 ± .062	.329	.574	.437
	Inverse Difference	.489 ± .065	.361	.617	.868	.503 ± .066	.373	.634	.959	.512 ± .064	.386	.637	.854
	Inverse Difference Moment	.495 ± .065	.368	.621	.935	.521 ± .066	.391	.652	.748	.531 ± .062	.409	.652	.622
	Inverse Difference Moment Normalized	.475 ± .065	.348	.601	.698	.492 ± .064	.366	.619	.906	.535 ± .068	.400	.669	.612
	Inverse Difference Normalized	.471 ± .065	.344	.598	.652	.496 ± .065	.369	.623	.952	.516 ± .067	.386	.647	.806
	Informational Measure of Correlation 1	.564 ± .067	.432	.695	.343	.508 ± .069	.373	.642	.912	.524 ± .067	.392	.656	.725
	Informational Measure of Correlation 2	.417 ± .068	.284	.550	.221	.478 ± .067	.347	.610	.745	.583 ± .068	.450	.716	.220
	Inverse Variance	.537 ± .061	.417	.658	.542	.515 ± .060	.398	.633	.798	.558 ± .058	.445	.671	.314
	Joint Average	.396 ± .062	.274	.517	.093	.405 ± .062	.284	.526	.123	.528 ± .063	.404	.652	.655
	Joint Energy	.497 ± .065	.370	.624	.963	.523 ± .066	.394	.651	.728	.520 ± .067	.389	.650	.769
	Joint Entropy	.510 ± .066	.381	.639	.880	.476 ± .067	.345	.606	.717	.536 ± .066	.405	.666	.592
	Maximal Correlation Coefficient	.367 ± .066	.239	.496	**.043**	.492 ± .070	.355	.629	.906	.635 ± .059	.519	.752	**.023**
	Maximum Probability	.517 ± .066	.387	.646	.802	.531 ± .063	.407	.655	.627	.489 ± .064	.363	.615	.866
	Sum Average	.410 ± .064	.285	.535	.156	.421 ± .063	.298	.545	.211	.544 ± .063	.420	.669	.484
	Sum Entropy	.423 ± .062	.302	.544	.211	.417 ± .063	.294	.541	.190	.531 ± .063	.408	.654	.620
	Sum Squares	.420 ± .068	.288	.553	.239	.487 ± .070	.349	.625	.858	.503 ± .067	.372	.634	.964
MUDM	Dependence Entropy	.534 ± .064	.408	.660	.600	.505 ± .065	.378	.631	.943	.532 ± .067	.400	.664	.635
	Dependence Non-Uniformity	.538 ± .067	.407	.669	.566	.486 ± .066	.357	.615	.834	.571 ± .069	.437	.706	.299
	Dependence Non-Uniformity Normalized	.420 ± .065	.292	.548	.219	.457 ± .067	.326	.589	.524	.570 ± .061	.450	.690	.252
	Dependence Variance	.608 ± .065	.482	.735	.093	.538 ± .065	.410	.666	.559	.600 ± .061	.481	.718	.101
	MU Non-Uniformity	.562 ± .067	.432	.693	.350	.514 ± .067	.382	.646	.835	.591 ± .066	.462	.720	.168
	MU Variance	.420 ± .066	.290	.549	.225	.461 ± .066	.331	.590	.552	.521 ± .068	.387	.654	.759
	High MU Emphasis	.401 ± .063	.279	.524	.115	.408 ± .062	.286	.529	.138	.514 ± .063	.391	.638	.818

	Large Dependence Emphasis	.589 ± .065	.462	.716	.167	.531 ± .066	.401	.661	.637	.599 ± .061	.480	.718	.103
	Large Dependence High MU Emphasis	.429 ± .063	.306	.553	.263	.403 ± .061	.283	.522	.111	.464 ± .065	.338	.591	.582
	Large Dependence Low MU Emphasis	.662 ± .062	.540	.783	**.009**	.567 ± .063	.445	.690	.282	.622 ± .061	.502	.741	**.045**
	Low MU Emphasis	.562 ± .066	.433	.691	.344	.531 ± .063	.408	.655	.618	.504 ± .065	.377	.630	.952
	Small Dependence Emphasis	.443 ± .065	.316	.570	.382	.474 ± .066	.345	.603	.690	.550 ± .063	.427	.673	.426
	Small Dependence High MU Emphasis	.451 ± .067	.319	.582	.462	.456 ± .066	.327	.585	.505	.506 ± .066	.378	.635	.925
	Small Dependence Low MU Emphasis	.498 ± .064	.373	.624	.980	.477 ± .063	.353	.602	.720	.590 ± .060	.474	.707	.129
MURLM	MU Non-Uniformity	.563 ± .067	.432	.694	.347	.514 ± .067	.383	.646	.829	.589 ± .066	.460	.719	.176
	MU Non-Uniformity Normalized	.579 ± .063	.456	.702	.208	.591 ± .061	.472	.711	.134	.562 ± .065	.434	.690	.342
	MU Variance	.418 ± .066	.289	.547	.214	.460 ± .067	.329	.591	.553	.525 ± .069	.390	.659	.720
	High MU Run Emphasis	.410 ± .063	.286	.534	.156	.414 ± .062	.291	.536	.168	.511 ± .064	.385	.637	.861
	Long Run Emphasis	.482 ± .062	.360	.604	.773	.509 ± .064	.384	.634	.886	.416 ± .058	.302	.530	.150
	Long Run High MU Emphasis	.407 ± .062	.286	.529	.135	.432 ± .063	.309	.555	.279	.481 ± .064	.356	.606	.765
	Long Run Low MU Emphasis	.585 ± .064	.460	.711	.184	.543 ± .063	.419	.666	.501	.554 ± .062	.433	.675	.381
	Low MU Run Emphasis	.560 ± .066	.430	.690	.362	.529 ± .063	.405	.653	.650	.495 ± .066	.366	.624	.936
	Run Entropy	.426 ± .062	.304	.548	.233	.422 ± .062	.301	.543	.204	.509 ± .065	.382	.635	.895
	Run Length Non-Uniformity	.547 ± .066	.417	.677	.478	.486 ± .067	.354	.618	.833	.578 ± .068	.446	.711	.246
	Run Length Non-Uniformity Normalized	.405 ± .065	.277	.532	.143	.452 ± .068	.319	.585	.476	.585 ± .066	.456	.714	.197
	Run Percentage	.404 ± .065	.276	.532	.142	.452 ± .068	.319	.585	.480	.583 ± .066	.454	.711	.209
	Run Variance	.505 ± .064	.379	.630	.943	.488 ± .063	.365	.610	.843	.416 ± .059	.301	.531	.152
	Short Run Emphasis	.405 ± .065	.277	.532	.144	.451 ± .068	.318	.584	.472	.583 ± .066	.454	.712	.206
	Short Run High MU Emphasis	.415 ± .064	.290	.540	.181	.411 ± .062	.290	.533	.155	.507 ± .064	.382	.631	.917
	Short Run Low MU Emphasis	.557 ± .066	.428	.687	.387	.530 ± .063	.406	.654	.635	.489 ± .066	.359	.618	.865
MUSZM	MU Non-Uniformity	.552 ± .067	.421	.682	.437	.502 ± .067	.370	.634	.978	.596 ± .068	.463	.729	.157
	MU Non-Uniformity Normalized	.574 ± .062	.453	.695	.229	.579 ± .061	.461	.698	.190	.652 ± .057	.541	.764	**.007**

	MU Variance	.438 ± .068	.305	.571	.362	.450 ± .065	.322	.578	.441	.508 ± .069	.373	.643	.907
	High MU Zone Emphasis	.424 ± .067	.293	.554	.253	.427 ± .066	.297	.557	.269	.505 ± .064	.380	.630	.940
	Large Area Emphasis	.576 ± .065	.449	.703	.239	.528 ± .066	.400	.656	.669	.568 ± .062	.448	.689	.268
	Large Area High MU Emphasis	.470 ± .064	.345	.595	.642	.432 ± .063	.308	.556	.285	.533 ± .062	.411	.655	.597
	Large Area Low MU Emphasis	.638 ± .062	.517	.759	**.026**	.568 ± .065	.441	.695	.293	.631 ± .064	.506	.757	**.041**
	Low MU Zone Emphasis	.519 ± .067	.387	.651	.777	.503 ± .064	.377	.628	.968	.627 ± .060	.509	.745	**.035**
	Size Zone Non-Uniformity	.539 ± .066	.409	.669	.559	.481 ± .065	.353	.609	.768	.595 ± .066	.465	.725	.150
	Size Zone Non-Uniformity Normalized	.470 ± .064	.345	.596	.644	.489 ± .064	.363	.614	.857	.544 ± .062	.423	.666	.474
	Small Area Emphasis	.474 ± .064	.350	.599	.688	.486 ± .064	.361	.610	.823	.537 ± .063	.413	.661	.558
	Small Area High MU Emphasis	.462 ± .069	.327	.597	.577	.457 ± .067	.327	.587	.518	.510 ± .066	.380	.640	.881
	Small Area Low MU Emphasis	.497 ± .064	.371	.624	.969	.493 ± .063	.369	.617	.910	.664 ± .056	.554	.775	**.004**
	Zone Entropy	.511 ± .064	.386	.636	.860	.493 ± .063	.370	.616	.912	.527 ± .065	.399	.656	.676
	Zone Percentage	.438 ± .065	.311	.566	.344	.469 ± .066	.340	.599	.643	.546 ± .064	.420	.672	.477
	Zone Variance	.585 ± .065	.457	.712	.193	.529 ± .065	.402	.656	.655	.593 ± .061	.473	.712	.128
NMUDM	Busyness	.583 ± .063	.459	.708	.188	.553 ± .063	.428	.677	.406	.587 ± .066	.459	.716	.184
	Coarseness	.452 ± .067	.320	.584	.476	.496 ± .065	.368	.624	.952	.560 ± .064	.434	.686	.352
	Complexity	.426 ± .064	.301	.552	.249	.470 ± .064	.345	.595	.636	.431 ± .064	.306	.556	.277
	Contrast	.449 ± .067	.318	.580	.443	.497 ± .067	.365	.628	.959	.532 ± .063	.408	.657	.609
	Strength	.441 ± .068	.308	.574	.387	.511 ± .068	.378	.644	.874	.541 ± .068	.408	.674	.544

Abbreviations: SUV, standardized uptake value; MTV, metabolic tumor volume; TLG, total lesion glycolysis; 95% CI, 95% confidence interval; AUC, area under the curve; SD, standard deviation; LB, lower bound; UB, upper bound; MU, metabolic uptake; RF_{50} or RF_{60}, diagnostic performance of radiomic features extracted from metabolic tumor volume, as measured on FDG PET/CT using thresholds of 50% and 60%, respectively; RF_{Diff}, diagnostic performance of the absolute value of the differences between radiomic features using thresholds of 50% and 60%

Table S2: Checklist for evaluation of Radiomics research.

Section	No.	Item	Yes	No	n/a	Page
**Title**						
	1	Relevant title, specifying the radiomic methodology	☐	☑	☐	
**Abstract**						
	2	Structured summary with relevant information	☑	☐	☐	1-2
**Keywords**						
	3	Relevant keywords for radiomics	☑	☐	☐	2
**Introduction**						
	4	Scientific or clinical background	☑	☐	☐	2-3
	5	Rationale for using a radiomic approach	☑	☐	☐	2-3
	6	Study objective(s)	☑	☐	☐	3
**Method**						
Study Design	7	Adherence to guidelines or checklists (e.g., CLEAR checklist)	☑	☐	☐	3
	8	Ethical details (e.g., approval, consent, data protection)	☑	☐	☐	3
	9	Sample size calculation	☐	☑	☐	
	10	Study nature (e.g., retrospective, prospective)	☑	☐	☐	3
	11	Eligibility criteria	☑	☐	☐	4
	12	Flowchart for technical pipeline	☑	☐	☐	Figure 1
Data	13	Data source (e.g., private, public)	☑	☐	☐	4
	14	Data overlap	☐	☐	☑	
	15	Data split methodology	☑	☐	☐	7
	16	Imaging protocol (i.e., image acquisition and processing)	☑	☐	☐	5
	17	Definition of non-radiomic predictor variables	☑	☐	☐	6
	18	Definition of the reference standard (i.e., outcome variable)	☑	☐	☐	3
Segmentation	19	Segmentation strategy	☑	☐	☐	6
	20	Details of operators performing segmentation	☐	☐	☑	
Pre-processing	21	Image pre-processing details	☐	☐	☑	
	22	Resampling method and its parameters	☐	☐	☑	
	23	Discretization method and its parameters	☑	☐	☐	6
	24	Image types (e.g., original, filtered, transformed)	☑	☐	☐	5
Feature extraction	25	Feature extraction method	☑	☐	☐	6
	26	Feature classes	☑	☐	☐	6
	27	Number of features	☑	☐	☐	6
	28	Default configuration statement for remaining parameters	☐	☐	☑	
Data preparation	29	Handling of missing data	☐	☐	☑	
	30	Details of class imbalance	☐	☐	☑	
	31	Details of segmentation reliability analysis	☐	☐	☑	
	32	Feature scaling details (e.g., normalization, standardization)	☐	☐	☑	
	33	Dimension reduction details	☐	☑	☐	
Modeling	34	Algorithm details	☑	☐	☐	5

	35	Training and tuning details	☐	☑	☐	
	36	Handling of confounders	☐	☑	☐	
	37	Model selection strategy	☑	☐	☐	7
Evaluation	38	Testing technique (e.g., internal, external)	☑	☐	☐	7
	39	Performance metrics and rationale for choosing	☑	☐	☐	7
	40	Uncertainty evaluation and measures (e.g., confidence intervals)	☐	☑	☐	
	41	Statistical performance comparison (e.g., DeLong's test)	☑	☐	☐	7
	42	Comparison with non-radiomic and combined methods	☐	☐	☑	
	43	Interpretability and explainability methods	☑	☐	☐	7
**Results**			☐	☐	☐	
	44	Baseline demographic and clinical characteristics	☑	☐	☐	Table 1
	45	Flowchart for eligibility criteria	☑	☐	☐	Figure 2
	46	Feature statistics (e.g., reproducibility, feature selection)	☑	☐	☐	Figure 5、6
	47	Model performance evaluation	☑	☐	☐	Table 2
	48	Comparison with non-radiomic and combined approaches	☐	☐	☑	
**Discussion**			☐	☐	☐	
	49	Overview of important findings	☑	☐	☐	12-13
	50	Previous works with differences from the current study	☑	☐	☐	13
	51	Practical implications	☑	☐	☐	13
	52	Strengths and limitations (e.g., bias and generalizability issues)	☑	☐	☐	13-14
**Open Science**						
Data availability	53	Sharing images along with segmentation data [n/e]	☐	☑	☐	
	54	Sharing radiomic feature data	☐	☑	☐	
Code availability	55	Sharing pre-processing scripts or settings	☐	☑	☐	
	56	Sharing source code for modeling	☐	☑	☐	
Model availability	57	Sharing final model files	☐	☑	☐	
	58	Sharing a ready-to-use system [n/e]	☐	☑	☐	

Yes, details provided; **No**, details not provided; **n/e**, not essential; **n/a**, not applicable
